# The Effect of Post-Exercise Cryotherapy on Recovery Characteristics: A Systematic Review and Meta-Analysis

**DOI:** 10.1371/journal.pone.0139028

**Published:** 2015-09-28

**Authors:** Erich Hohenauer, Jan Taeymans, Jean-Pierre Baeyens, Peter Clarys, Ron Clijsen

**Affiliations:** 1 Department of Business Economics, Health and Social Care, University of Applied Sciences and Arts of Southern Switzerland, Landquart / Manno, Switzerland; 2 Health Department, Bern University of Applied Sciences, Berne, Switzerland; 3 Faculty of Physical Education and Physiotherapy, Vrije Universiteit Brussel, Brussels, Belgium; 4 University College Physiotherapy Thim van der Laan, Landquart (GR), Switzerland; 5 Faculty of Medicine and Health Sciences, University of Antwerp, Antwerp, Belgium; 6 Faculty of Applied Engineering, University of Antwerp, Antwerp, Belgium; West Virginia University School of Medicine, UNITED STATES

## Abstract

The aim of this review and meta-analysis was to critically determine the possible effects of different cooling applications, compared to non-cooling, passive post-exercise strategies, on recovery characteristics after various, exhaustive exercise protocols up to 96 hours (hrs). A total of n = 36 articles were processed in this study. To establish the research question, the PICO-model, according to the PRISMA guidelines was used. The Cochrane’s risk of bias tool, which was used for the quality assessment, demonstrated a high risk of performance bias and detection bias. Meta-analyses of subjective characteristics, such as delayed-onset muscle soreness (DOMS) and ratings of perceived exertion (RPE) and objective characteristics like blood plasma markers and blood plasma cytokines, were performed. Pooled data from 27 articles revealed, that cooling and especially cold water immersions affected the symptoms of DOMS significantly, compared to the control conditions after 24 hrs recovery, with a standardized mean difference (Hedges’ g) of -0.75 with a 95% confidence interval (CI) of -1.20 to -0.30. This effect remained significant after 48 hrs (Hedges’ g: -0.73, 95% CI: -1.20 to -0.26) and 96 hrs (Hedges’ g: -0.71, 95% CI: -1.10 to -0.33). A significant difference in lowering the symptoms of RPE could only be observed after 24 hrs of recovery, favouring cooling compared to the control conditions (Hedges’ g: -0.95, 95% CI: -1.89 to -0.00). There was no evidence, that cooling affects any objective recovery variable in a significant way during a 96 hrs recovery period.

## Introduction

For decades, different cryotherapies–such as cold-water immersions (CWI) and ice packs–have been used for post-exercise recovery in a variety of sports to cope with fatigue and/or delayed-onset muscle soreness (DOMS) [1]. Exercising at different intensity levels induces various degrees of fatigue to the musculoskeletal, nervous and metabolic systems [2]. Exercise is also associated with microscopic tears in the muscle tissue, commonly described as exercise induced muscle damage, leading to DOMS [3].

In sports medicine, cryotherapy, as a post-exercise intervention, has been investigated by means of subjective ratings of DOMS and general ratings of perceived exertion (RPE), objective attenuations of blood plasma markers such as creatine-kinase (CK) and lactate-levels, or blood plasma cytokines encompassing Interleukines (IL) and C-reactive protein (CRP) [1, 2, 4–37]. Cryotherapy is commonly described as a procedure to relieve pain and to decrease inflammation in musculoskeletal problems [38]. The mechanism of cold therapy for recovery after exercise is predominantly attributed to its vasoconstrictive effect, which reduces the inflammation reactions through a decrease of the cell metabolism [39]. In sports, cooling represents a considerable therapy for athletes. A recently published meta-analysis from Leeder et al. (2012) showed that CWI is an effective strategy to reduce the symptoms of DOMS following a range of strenuous and exhaustive exercise types [40]. These results are in line with those of Bleakley et al. (2012). These authors recently demonstrated significant differences in their meta-analysis, favouring cooling compared to passive interventions up to 96 hrs [3]. In their review article, Banfi et al. (2010) recently indicated, that whole-body cooling (WBC) is a possible application to treat muscle injuries, muscle syndromes of overuse and to reduce the recovery time between training sessions. The WBC treatment did not induce modifications of biochemical and haematological parameters. The authors concluded that WBC is not harmful and does not induce general or specific negative effects in athletes [38]. However, the negative effects of cooling and the area of application must also be critically examined. Takagi et al. (2011) recently showed, that applying ice bags for 20 minutes (min) after a crush injury to the extensor digitorum longus muscle in rats, led to a delay in muscle regeneration, impairment of muscle regeneration and redundant collagen synthesis due to reduced calpain activity. This enzyme is necessary for the primary reaction of muscle-degeneration. A decreased number of macrophages and suppressed inflammation after local cooling applications might be closely related to the retarded healing processes [41–43]. The effects of post-exercise cryotherapy on recovery dependent variables, such as DOMS, blood plasma markers, blood plasma cytokines, different performance parameters and RPE in non-injured participants are still unclear. A lack of appropriate recovery may as a result prevent the athlete of training at a required intensity or, moreover, of achieving the required load during the consecutive training session(s). Furthermore, full recovery is necessary for optimal competition performance [44]. The aim of this study was to critically determine the effects of different cooling applications compared to non-cooling, passive post-exercise strategies by means of recovery characteristics after various exhaustive exercise protocols up to 96 hours.

### Research Question and Search Algorithm

The research question was defined by the PICO-model in accordance with the Preferred Reporting Items for Systematic Reviews and Meta-Analyses (PRISMA) statement [45]: Population: healthy, non-injured, female and male study participants, participants could be of any training status; Intervention: external applications, only post-exercise cryotherapy accepted; Comparator: passive control interventions without cooling, passive sham / placebo applications; Outcomes: DOMS and RPE, levels of venous or capillary blood plasma markers (CK-levels and blood/muscle lactate-levels respectively lactate dehydrogenase [LDH]-levels) and/or blood plasma cytokines (IL-6, CRP), measured 24 hrs, 48 hrs, 72 hrs and/or 96 hrs after exhaustive exercise. DOMS and RPE were defined as subjective recovery characteristics in this study. The subjective perception of DOMS was rated on various scaling systems, such as visual analogue scales (VAS), BORG scales or Likert-scales. RPE was rated by various scaling systems, such as BORG scales or VAS. CK-levels, lactate-levels, CRP and IL-6 were defined as objective recovery characteristics in this study. The rationale for inclusion of these outcome variables was comparability. Numerous researchers who tried to identify a subjective and objective recovery profile after exhaustive exercises previously used these variables in their studies.

## Methods

### Literature Search Strategies and Data Sources

A systematic search was accomplished electronically between October 2013 and August 2014 in the MEDLINE (PubMed), SportDiscus and PEDro databases according to the PRISMA statement [45]. The following key words and their combinations were used without using any automatic filters: “Exercise AND Cold OR Cooling OR Cryotherapy AND Recovery OR Recovery Strategy OR Recovery Modality”. Seven additional articles were included after having read the reference list of the eligible studies (n = 49).

### Study Selection Criteria

**Fig 1:**
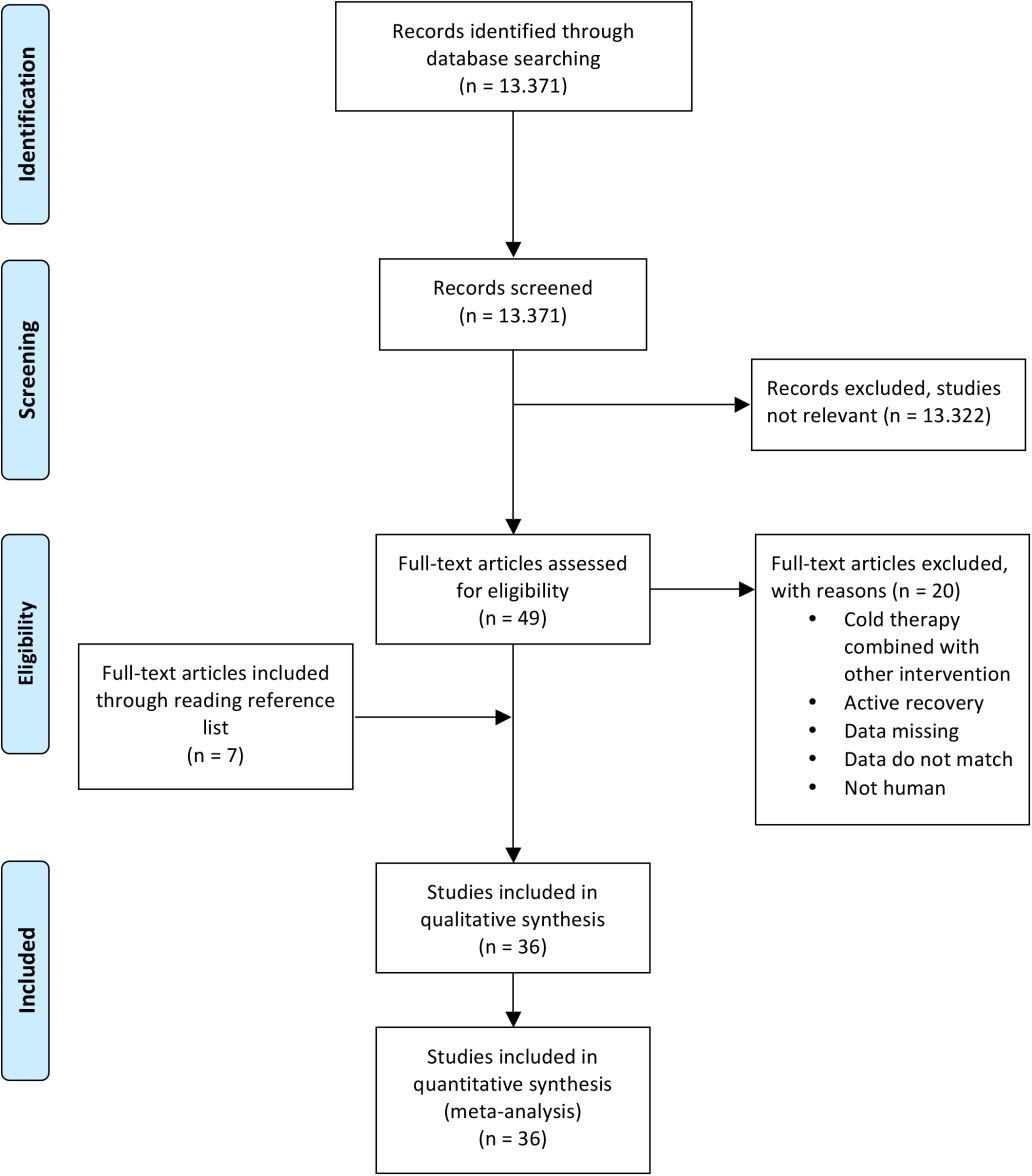
Flow-chart describing the systematic review procedure.

Inclusion criteria for the articles were: (1) the study design was randomized or quasi randomized into an intervention group and a non-cooling, passive control group; (2) the cold therapy had to be an external application form without combinations, focused on the mentioned outcome variables after the exercise bouts; (3) the cold interventions had to be compared to at least one control condition; (4) the outcome variables had to be measured immediately (0–24 hrs), and / or 24 hrs, and / or 48 hrs, and / or 72 hrs and/or 96 hrs post-exercise; (5) the data of the outcome variables had to be processed in the articles; (6) the human volunteers had to be healthy without any physical infirmity; (7) the human volunteers could be of any athletic training status; (8) there were no sex-defined inclusion or exclusion criteria; (9) at least one of the subjective or objective recovery characteristics had to be evaluated in the articles; (10) the cold therapy had to be applied within one hour after the end of the exercise protocol; (11) all exhaustive exercise types or muscle damaging protocols were accepted; (12) English and German language restrictions. Articles, which did not meet these criteria were excluded from this study. Fig 1 shows the systematic search strategy and selection process.

### Data Analysis

General characteristics of the studies and results of the individual studies were extracted independently by two researchers (EH, RC). In case of a lack of information, the statistics were extrapolated from the given figures and graphs. In case of disagreement, consensus was sought by involving a third researcher (JPB). Meta-analyses with a random-effects model (specified a priori), allowing for possible between–studies heterogeneity, were used to examine the overall effect size of cooling applications on the subjective and objective recovery characteristics. The inversed variance method was used to calculate the weighting factors for the included, individual study results. The different individual study effect sizes were standardized using Cohen’s d, by dividing the effect sizes by the pooled standard deviations, as proposed by Borenstein et al. (2009) [46]. The effect sizes were expressed as Hedges’ g, to account for possible overestimation of the true population effect size in small studies. The magnitude of “g” was interpreted according to the following scale [47]: < .20 = negligible effect, .20 - .49 = small effect, .50 - .79 = moderate effect, ≥ .80 = large effect. The Cochran’s Q statistics and its corresponding p-Value, as well as the I^2^,were calculated to assess across studies heterogeneity and its degree respectively [48]. If between-study heterogeneity was observed, subgroup analyses or univariate meta-regression analyses were performed to assess the potential influence of different co-variates, such as cooling modality (CWI, WBC, cold air, cold pack), sex (discrete data) and cooling temperature (continuous data) on the pooled effect size. Two of the 36 studies used extreme low cooling temperatures (up to -110°C) [1, 8]. To assess the robustness of the overall weighted estimate against these extreme cooling temperatures, a sensitivity analysis was conducted. Publication bias was assessed using visual analysis of the funnel plot and formal testing for funnel plot asymmetries using the “trim and fill” and “fail and safe” algorithms [46]. The Comprehensive Meta-Analysis 2 software (CMA- Version 2 Professional, Biostat Inc., Englewood, USA) was used for the calculations of the weighted overall estimates, the corresponding 95% confidence intervals (95% CI) and to establish the forest plots.

### Data Synthesis

In this article the subjective variables under investigation were DOMS and RPE. The objective outcomes of interest for the meta-analyses were blood plasma markers (CK, blood/muscle lactate, LDH) and blood plasma cytokines (IL-6, CRP). Meta-analyses were conducted using means ± standard deviations (SD) or standard errors (SE).

### Risk of Bias

The systematic error of the 36 articles was assessed using the Cochrane’s risk-of-bias tool. [49]. Two researchers (EH, RC) independently scored each trial for the risk of bias. In case of disagreement, a third researcher (PC) rated the questionable item and agreement was sought by consensus. Each study was graded for the following domains: random sequence generation, allocation concealment, blinding participants, blinding personnel, blinding outcome assessors, incomplete outcome data, selective reporting and other bias. They were rated “low” (+) if the risk of bias for this item was low, or “high” (-) if the risk of bias for this item was high. In case of insufficient reported information, or information which made an interpretation questionable and thus unclear, the risk of bias item was rated as “unclear” (?).

## Results

### Risk of Bias Analysis

**Fig 2:**
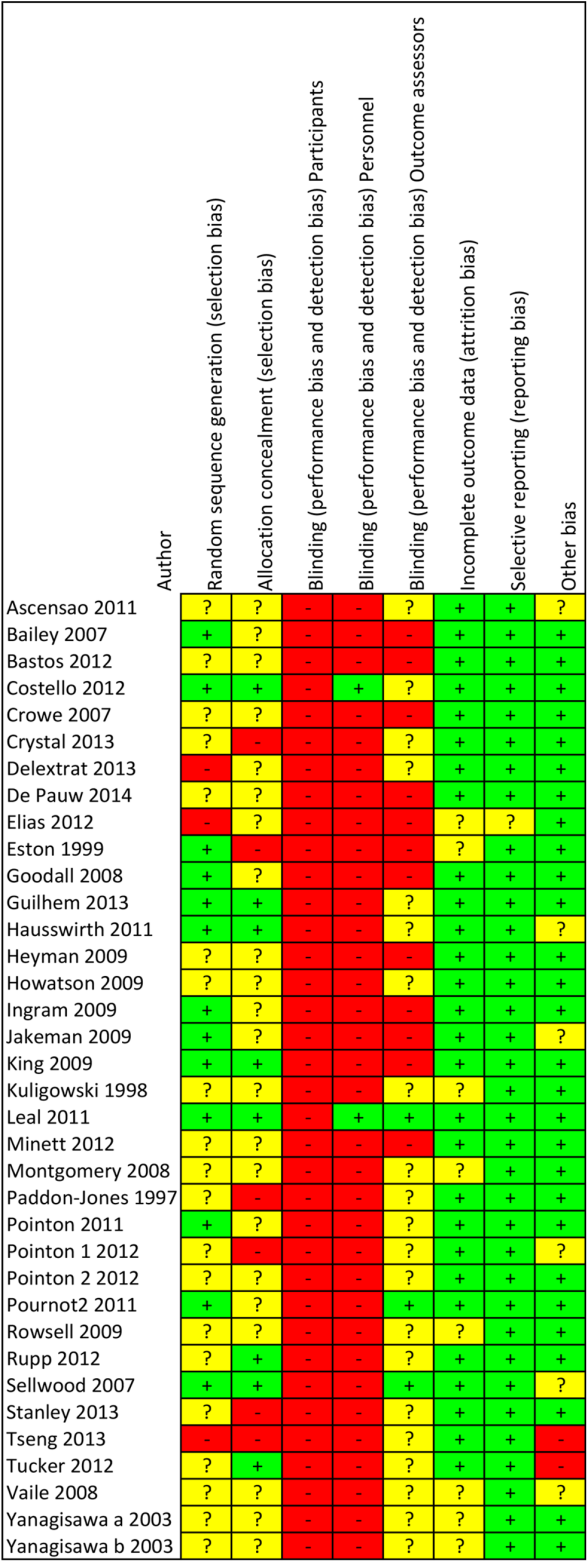
Risk of bias graph for each included study.

**Fig 3:**
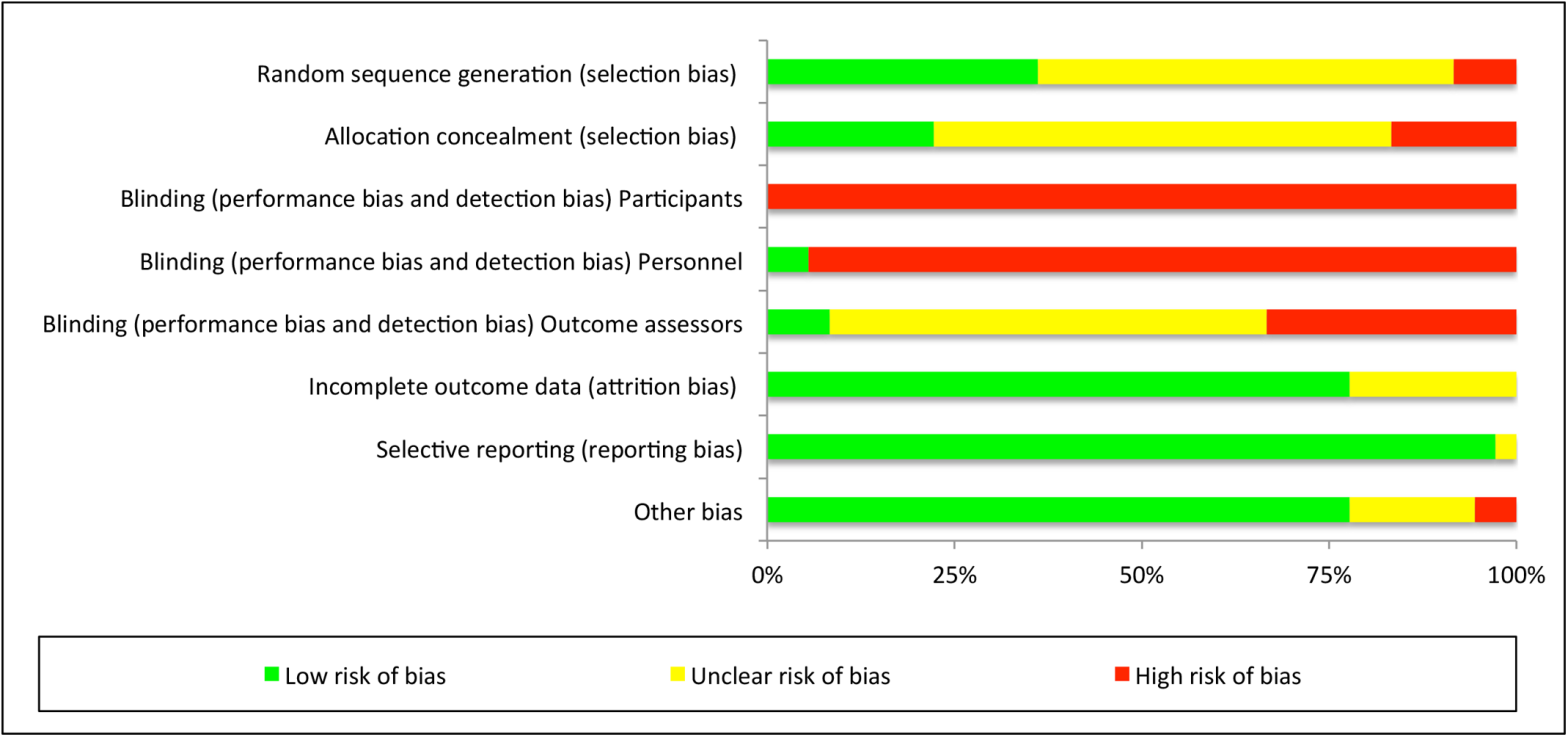
Risk of bias summary for all included studies.

The risk of bias analysis demonstrated a high risk of bias for the blinding procedures. Blinding of the participants was only described in two studies [1, 9]. Personnel blinding was also described in two studies [9, 30] only. Blinding of outcome assessors was only found in two studies [29, 31]. The selection bias ratings stayed unclear because of insufficient or unclear information. A low risk of reporting bias and other bias could be observed throughout the studies. The full details of the risk of bias analysis for all and individual single studies can be observed in Figs 2 and 3.

### Study and Population Characteristics

A total of n = 36 articles met the inclusion criteria. These trials were processed in the present article as sources of primary research data, while the systematic review articles and meta-analyses were used in the introduction and the discussion part. Characteristics of the included studies are summarized in Table 1. The total study population of all selected articles comprised 574 healthy volunteers with an unequal distribution of sex. The selected articles comprised 412 male and 72 female volunteers. In three articles, with a total population of 90 male and female volunteers, it was not possible to identify the sex distribution [23, 30, 31]. Four studies included only female volunteers [6, 18, 22, 50], while two studies included males predominantly [1, 5]. In one study, female and male were equally distributed [16]. Throughout all studies, the mean sample size was 16 volunteers (range = 6 to 40 volunteers) [9, 31]. The mean age of the total study population was 22 years (yrs). In one article, the age of the study population was not mentioned [33]. There were 18 randomized controlled trials [1, 4, 6, 7, 10, 12, 14, 15, 18, 19, 23, 25, 26, 29–31, 34, 35] and 18 randomized crossover trials [2, 5, 8, 9, 11, 16, 17, 20–22, 24, 27, 28, 32, 33, 36, 37, 50]. The experimental trials were conducted in Belgium (n = 2), France (n = 3), Ireland (n = 1), Portugal (n = 1), United Kingdom (n = 6), Australia (n = 14), Brazil (n = 2), United States (n = 4), Taiwan (n = 1) and Japan (n = 2). Eight studies involved well-trained/elite volunteers [4, 7–9, 17, 29, 30, 50]. Six of these studies included soccer / football players [4, 7, 9, 17, 29, 30]. One study worked with well-trained male runners [8] and another study chose well-trained female climbers [50]. The remaining 28 studies used healthy / trained volunteers [1, 2, 5, 6, 10–12, 14–16, 18–28, 31–37]. Detailed information about the training status can be observed in Table 1.

**Table 1:** Summary of the used studies for the meta-analysis.

A							
**Author / Country**	**Design / Publication year**	**Participant cohort (training status, sex, age)**	**Sample Size (n)**	**Experimental condition and temperature vs. control condition**	**Total cooling time (min)**	**Exercise Intervention**	**Outcome variables and time of measurement post exercise (hrs)**
Ascensao et al. [4] / POR	RCT / 2011	Male nat. jr. soccer players (18.1 ± 1.8 yrs exp., 18.3 ± 0.8 yrs control)	n = 20	CWI (10°C) vs. TNI (35°C)	10	One-off soccer match	DOMS (24 48)
			n = 10 exp.				CK (24 48)
			n = 10 con.				CRP (24 48)
Bailey et al. [14] / UK	RCT / 2007	Male habitually active (22.3 ± 3.3 yrs)	n = 20	CWI (10°C) vs. passive rest	10	Loughborough intermittent shuttle test	DOMS (24 48)
			n = 10 exp.				CK (24 48)
			n = 10 con.				
Bastos et al. [36] / BRA	RCO / 2012	Male active subjects (21 ± 2 yrs)	n = 20	CWI (11 ± 2°C) vs. passive rest	6	Cycling at constant velocity until exhaustion	BL (<24)
			n = 20 exp.				
			n = 20 con.				
Costello et al. [1] / IRL	RCT / 2012	Healthy volunteers 4f & 14m (21.2 ± 2.1 yrs)	n = 36	WBC (-60°C & -110°C) vs. passive rest	6	100 high-force maximal ecc. contractions of the knee extensors	DOMS (24 48 72 96)
			n = 9 exp.				
			n = 9 con.				
Crowe et al. [5] / AUS	RCO / 2007	Healthy volunteers 4f & 13m (21.5 ± 1.3 yrs)	n = 17	CWI (13–14°C) vs. passive rest	15	2 x 30 s "all out" cycling test within 60 min	BL (<24)
			n = 17 exp.				
			n = 17 con.				
Crystal et al. [15] / USA	RCT / 2013	Male healthy active volunteers (21.2 ± 2.3 yrs)	n = 20	CWI (3–5°C) vs. passive rest	20	40 min downhill treadmill run at -10% grade at 60% VO2max	DOMS (24 48 72)
			n = 10 exp.				
			n = 10 con.				
Delextrat et al. [16] / UK	RCO / 2013	Basketball players 8m (23 ± 3 yrs) & 8f (22 ± 2 yrs)	n = 16	CWI (11°C) vs. passive rest	10	Competitive basketball match	DOMS (24)
			n = 8 exp.				RPE (24)
			n = 8 con.				
B							
De Pauw et al. [37] / BEL	RCO / 2014	Male trained volunteers (22 ± 3 yrs)	n = 9	CWI (15°C) vs. passive rest	15	Cycle time trial (trial 1: 30 min; trial 2: 12 min)	BL (<24)
			n = 9 exp.				
			n = 9 con.				
Elias et al. [17] / AUS	RCO / 2012	Male professional Australian football players (20.9 ± 3.3 yrs)	n = 14	CWI (12°C) vs. passive rest	14	Australian football training	DOMS (24 48)
			n = 14 exp.				RPE (24 48)
			n = 14 con.				
Eston et al. [18] / UK	RCT / 1999	Female healthy volunteers (22.0 ± 2.9 yrs)	n = 15	CWI (15°C) vs. passive rest	15	8 x 5 ecc. upper arm flexions and extensions	DOMS (24 48 72)
			n = 8 exp.				CK (24 48 72)
			n = 7 con.				
Goodall et al. [19] / UK	RCT / 2008	Male physically active (24 ± 5 yrs)	n = 18	CWI (15°C) vs. passive rest	12	5 x 20 drop jumps	DOMS (24 48 72 96)
			n = 9 exp.				CK (24 48 72 96)
			n = 9 con.				
Guilhem et al. [10] / FRA	RCT / 2013	Male healthy volunteers (25.2 ± 1.1 yrs in exp., 23.9 ± 1.4 yrs in con.)	n = 24	Cold air (-30°C) vs. passive rest	12	3x 20 maximal isokinetic ecc. contractions of the elbow flexors	DOMS (24 48 72)
			n = 12 exp.				CK (24 48 72)
			n = 12 con.				IL-6 (24 48 72)
							CRP (24 48 72)
Hausswirth et al. [8] / FRA	RCO / 2011	Male well trained runners (31.8 ± 6.5 yrs)	n = 9	WBC (-10°C & -60°C & -110°C) vs. passive rest	9	48 min time running trail on 3 non-adjoining days	DOMS (24 48)
			n = 9 exp.				CK (24 48)
			n = 9 con.				RPE (24 48)
Heyman et al. [50] / BEL	RCO / 2009	Female well trained climbers (27.1 ± 8.9 yrs)	n = 13	CWI (15°C) vs. passive rest	15	Climbing until exhaustion	BL (<24)
			n = 13 exp.				
			n = 13 con.				
Howatson et al. [20] / UK	RCO / 2009	Male physically active volunteers (23 ± 3 yrs)	n = 16	CWI (15°C) vs. passive rest	12	5 x 20 drop jumps	DOMS (24 48 72 96)
			n = 16 exp.				CK (24 48 72 96)
			n = 16 con.				
C							
Ingram et al. [21] / AUS	RCO / 2009	Male athletes (27.5 ± 6.0 yrs)	n = 11	CWI (10°C) vs. passive rest	10	80 min of simulated team sport exercises	DOMS (24 48)
			n = 11 exp.				CK (24 48)
			n = 11 con.				CRP (24 48)
Jakeman et al. [6] / UK	RCT / 2009	Female physically active (19.9 ± 0.97 yrs)	n = 18	CWI (10°C) vs. passive rest	10	10 sets of 10 countermovement jumps within 10 min	DOMS (24 48 72 96)
			n = 9 exp.				CK (24 48 72 96)
			n = 9 con.				
King et al. [22] / AUS	RCO / 2009	Female trained netball players (19.5 ± 1.5 yrs)	n = 10	CWI (9.3°C) vs. passive rest (15 min)	10	Simulated netball match	DOMS (24)
			n = 10 exp.				BL (24)
			n = 10 con.				
Kuligowski et al. [23] / USA	RCT / 1998	Healthy volunteers, 28m (21.1 ± 3.1 yrs) 28f (20.1 ± 2.1 yrs)	n = 56	CWI (12.8C) vs. passive rest	24	50 ecc. contractions of the elbow flexors	DOMS (24 48 72 96)
			n = 14 exp.				
			n = 14 con.				

Values are means ± SD; CWI = cold water immersion, TNI = thermoneutral immersion, exp. = experimental, con. = control, nat. jr. = national junior, f = female, m = male, ecc. = eccentric, conc. = concentric, DOMS = delayed-onset muscle soreness, RPE = ratings of perceived exhaustion, CK = creatine-kinase, BL = blood lactate, ML = muscle lactate, LDH = lactate dehydrogenase, IL-6 = interleukine-6, CRP = C-reactive protein.

### Characteristics of the Exercise Protocols

The exercise protocols encompassed soccer-games or tournaments [4, 7]. Six authors used cycle ergometer tests [5, 9, 12, 32, 36, 37]. Two of these cycle tests were sprint tests, while the remaining four were endurance tests [5, 9]. A total of n = 14 studies used functional exercises to create the pre-cooling load. This involved ten sets of ten counter movement jumps within ten min [6], 100 eccentric contractions of the knee extensors in a 20 set design [1], six sets of 25 maximal concentric and eccentric contractions for the knee extensors [2], five sets of ten eccentric contractions of the knee extensors [31], seven sets of ten eccentric knee extensions on a leg press [33], five sets of twelve respectively 20 eccentric ankle plantar flexion [34, 35] five sets of 20 drop jumps [19, 20], eight sets of five maximal eccentric contractions of the upper arm flexors, respectively extensors [18], 50 eccentric contractions of the elbow flexors [23], eight sets of eight eccentric contractions of the elbow flexors [26], three sets of maximum (max) 20 eccentric contractions of the elbow flexors with a three min passive recovery period between the sets and six sets of five eccentric elbow flexor contractions [10, 11]. Furthermore, the exercise protocols encompassed various endurance running protocols [8, 14, 15] and sprint running protocols [27, 28]. Other authors used sport specific training or match exercises. These involved basketball [16, 25], Australian football [17], climbing [50], netball [22], bowling [24], simulated team sport exercises [21] and exhaustive intermittent exercise tasks [29]. Table 1 gives a detailed overview of the conducted exercise protocols.

### Characteristics of the Cooling Applications

In the present article, the most common cold therapy application (28 studies) was CWI of the legs [4–7, 9, 14–23, 25, 27–37, 50]. Water temperature in the leg immersing studies was 5°C to 10°C in 15 articles [4, 6, 7, 9, 14, 15, 21, 22, 27–29, 31, 32, 34, 35] and 11°C to 15°C in 13 articles [5, 16–20, 23, 25, 30, 33, 36, 37, 50]. One author used an arm immersing protocol with a water temperature of 5°C [26] instead of a leg immersing protocol.

WBC with sequential entering -110°C, -60°C and -10°C cold chambers was used in two studies [1, 8]. One author used a cold air application of -30°C [10]. Local ice cuff/ cold pack or ice bag applications were used in three studies [2, 11, 12]. Pointon et al. (2011) used ice cuffs with 0.5°C, which covered the entire surface of the exercised leg [2]. Tseng et al. (2013) and Tucker et al. (2012) did not mention the temperature of their cold packs / ice bags, which covered the exercised legs [11, 12]. One author used a mixed method WBC application, comprised of a towel soaked in cold water (5°C) and worn over the head, neck and shoulders. Additionally, the participants had to wear an ice-vest and received ice packs, which were placed on the quadriceps and the hamstrings muscles. The ice-vest and ice packs were kept frozen at -20°C before application [24].

### Characteristics of the Passive Control Interventions

The predominant passive control condition, used in 30 studies, was sitting, standing or resting supine in a room with comfortable temperatures, ranging from 15°C to 24°C [1, 2, 5, 6, 8, 10, 12, 14–24, 26–30, 32–37, 50]. Another passive control condition, used in three studies, was the use of a thermoneutral immersion (TNI). The volunteers who received this control condition were immersed until the same levels, as described in the experimental group. Ascensao et al. (2011) submerged the volunteers to the iliac crest, Rowsell et al. (2009) to the mesosternal level and Sellwood et al. (2007) to the anterior iliac crest [4, 7, 31]. Leal et al. (2011) and Tseng et al. (2013) used a passive sham and placebo application as their control condition [9, 11]. Leal et al. (2011) used a placebo light emitting diode therapy (PLEDT) as their control condition [9]. Tseng et al. (2013) used a sham application for their control group [11]. Montgomery et al. (2008) used carbohydrate intake and a stretching protocol for the control group [25]. The volunteers were directed through a standardized programme of ten stretches completed twice for 15 s to the legs and the lower back bilaterally, and consumed a carbohydrate (1 g κγ body mass^-1^) snack, a carbohydrate bar (Powerbar^TM^) and 600 ml of fluid in form of a sports drink (Gatorade^TM^). We decided not to exclude this study because of the stretching protocol. In all studies the control and experimental groups received interventions of the same average duration.

### Cooling Effects on Subjective Recovery Characteristics

#### Cooling Effects on DOMS

We obtained the data from 27 articles which revealed the effectiveness of cooling on DOMS [1, 2, 4, 6, 8, 10, 11, 14–27, 29–32, 34, 35].

**Fig 4:**
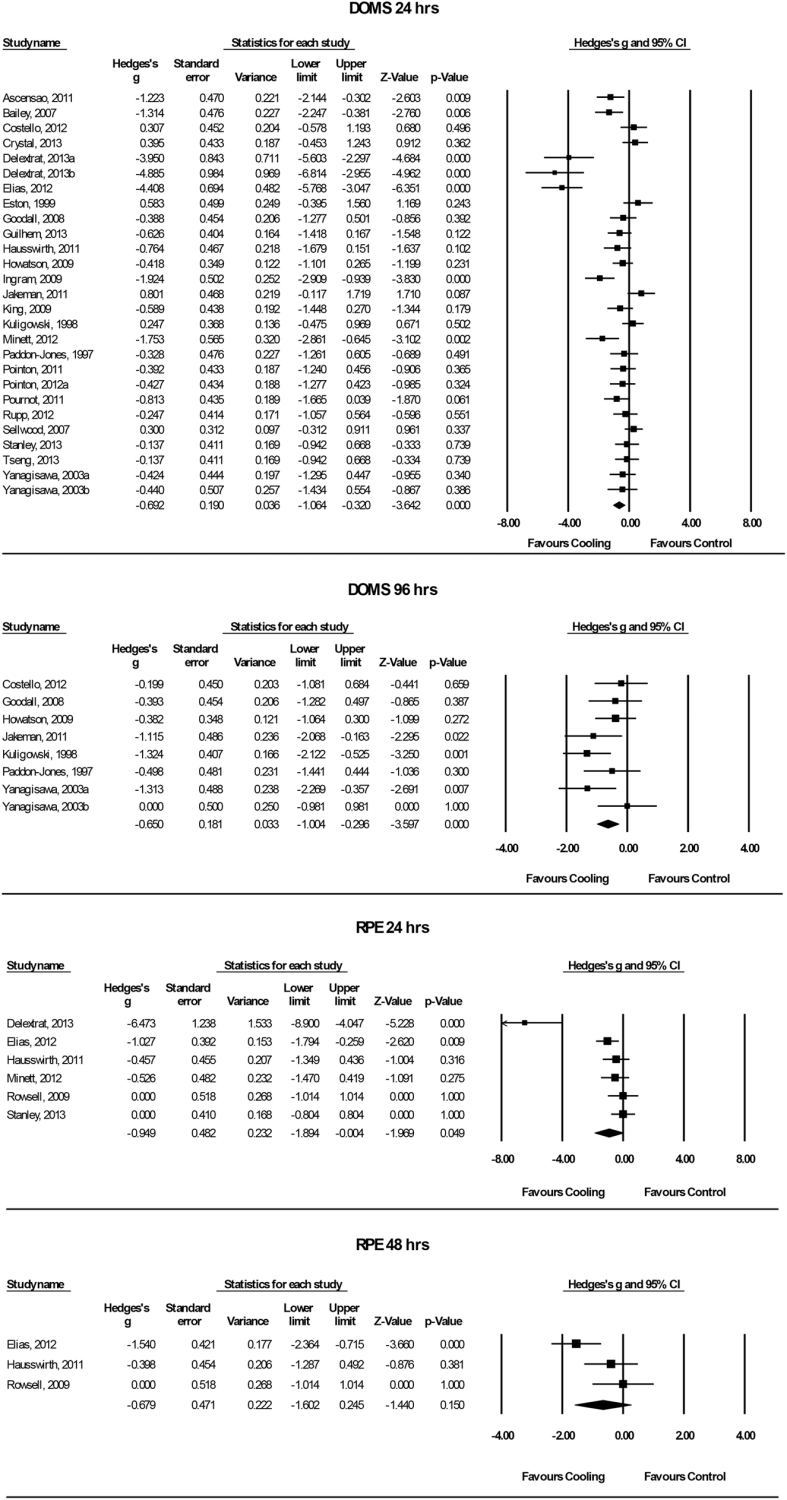
Forest plot of the meta-analysis illustrating the comparison of cooling versus control for measurement of DOMS AND RPE. DOMS = delayed-onset muscle soreness, RPE = ratings of perceived exertion.

Only two of these studies used a pain pressure algometer to measure DOMS [18, 26]. Six articles used a scale rating procedure, ranging from 0–10 [2, 4, 6, 10, 24, 29], one study a 0–50 scale [1], one study a 0–7 scale [11], one study a 1–12 scale [23], three studies a 1–10 scale [14, 25, 32] and two studies a 1–5 scale [34, 35]. Three studies used a 10-point Likert scale for their assessment [21, 22, 27]. Six studies used a 100 mm scale [8, 15–17, 30, 31] and two studies used a 200 mm scale [19, 20]. All of the above mentioned 27 articles measured the effect of cooling–compared to the control condition for DOMS–at least 24 hrs after the cooling application. The pooled results are presented in four subcategories, based on the follow-up time. A meta-analysis from 24 hrs up to 96 hrs could be performed for DOMS. Looking at the results in Fig 4, one can observe that cooling lowered the symptoms of DOMS 24 hrs after the application significantly compared to the control conditions (Hedges’ g: -0.69, 95% CI: -1.06 to -0.32; Cochran’s Q: 120.3, df (Q): 26, p < 0.001; I^2^: 78.4%). The classic fail and safe model analysis showed, that 317 negative studies would be needed before alpha increases above the 5% level for DOMS, measured 24 hrs after the cold application, indicating a low risk of publication bias. Cold therapy continued to attenuate the symptoms of DOMS significantly up to 96 hrs (48 hrs: Hedges’ g: -0.62, 95% CI: -1.00 to -0.25; Cochran’s Q: 71.2, df (Q): 19, p < 0.001; I^2^: 73.3%); (72 hrs: Hedges’ g: -0.18, 95% CI: -0.53 to 0.17; Cochran’s Q: 24.0, df (Q): 11, p = 0.013; I^2^: 54.3%); (96 hrs: Hedges’ g: -0.65, 95% CI: -1.00 to -0.30; Cochran’s Q: 9.2, df (Q): 7, p = 0.238; I^2^: 24.0%). Up to 72 hrs, a moderate to very high heterogeneity was observed [48]. The subgroup analysis according to different cooling modalities, sex or cooling temperature decreased the heterogeneity somewhat, but could not significantly explain it. The subgroup analysis for different cooling modalities showed that CWI significantly revealed reduced DOMS compared to cold air, cold pack and WBC up to 96 hrs (24 hrs: Hedges’ g: -0.75, 95% CI: -1.20 to -0.30; Cochran’s Q: 110.9, df (Q): 20, p < 0.001; I^2^: 82.0%); (48 hrs: Hedges’ g: -0.73, 95% CI: -1.20 to -0.26; Cochran’s Q: 63.3, df (Q): 14, p < 0.001; I^2^: 77.9%); (72 hrs: Hedges’ g: -0.32, 95% CI: -0.74 to 0.10; Cochran’s Q: 18.4 df (Q): 8, p = 0.018; I^2^: 56.7%); (96 hrs: Hedges’ g: -0.71, 95% CI: -1.10 to -0.33; Cochran’s Q: 8.1, df (Q): 6, p = 0.233; I^2^: 25.7%). These results indicate, that the findings in favour of cooling are dependent on the cooling modality. Subgroup analysis according to sex showed significant different effects, favouring cooling for male participants compared to female participants, up to 48 hrs (24 hrs: Hedges’ g: -0.92, 95% CI: -1.37 to -0.48; Cochran’s Q: 72.4, df (Q): 18, p < 0.001; I^2^: 75.2%); (48 hrs: Hedges’ g: -0.76, 95% CI: -1.21 to -0.32; Cochran’s Q: 47.6, df (Q): 16, p < 0.001; I^2^: 66.9%). To analyse the effect of the different cooling temperatures on the attenuation of the symptoms of DOMS, a meta-regression analysis between these parameters was performed. The meta-regression for the cooling temperature across the recovery time showed no significant correlation between cooling temperature and relieving symptoms of DOMS (48 hrs: Slope = -0.001, 95% CI: -0.007 to 0.004, p = 0.532; 72 hrs: Slope = -0.005, 95% CI: -0.01 to 0.00, p = 0.148). Visual analysis of the regression showed two extreme low temperatures used in the study from Hausswirth et al. (2011) and Costello et al. (2012) [1, 8]. These two authors used cold chambers with the coldest chamber set at -110°C. After excluding these two authors for the sensitivity analysis, the results still did not correlate significantly after performing the meta-regression (48 hrs: Slope = 0.002, 95% CI: -0.02 to 0.02, p = 0.78; 72 hrs: Slope = -0.005, 95% CI: -0.03 to 0.01, p = 0.552; 96 hrs: Slope = 0.007, 95% CI: -0.07 to 0.08, p = 0.854).

#### Cooling Effects on RPE

In this composed article, data of n = 6 studies were pooled up to 48 hrs for the analysis of the subjective RPE [7, 8, 16, 17, 24, 32]. The authors used various rating systems, such as 0–10 scales [24], 0–100 mm scales [8, 16], 1–10 scales [17, 32] and 6–20 scales [7]. The subjective ratings of perceived exertion demonstrated significantly different results favouring cooling compared to the control condition after 24 hrs of recovery (Hedges’ g: -0.95, 95% CI: -1.89 to -0.00; Cochran’s Q: 27.3, df (Q): 5, p < 0.001; I^2^: 81.7%). The meta-regression according to cooling temperature could not explain this high heterogeneity, nor show a significant correlation between cooling temperature and Hedges’ g (Slope = -0.001, 95% CI: 0.009 to 0.006, p = 0.707). Two cooling modalities (CWI: n = 4, WBC: n = 2) were used to assess the effect of cooling on RPE. The subgroup analysis according to cooling modality showed no significant differences between the cooling modalities, measured after 24 hrs of recovery (Cochran’s Q: 0.51, df (Q): 1, p = 0.477). No significant differences between cooling and the control condition could be observed after 48 hrs of recovery. (Hedges’ g: -0.68, 95% CI: -1.60 to 0.25; Cochran’s Q: 6.2, df (Q): 2, p = 0.044; I^2^: 67.9%).

### Cooling Effects on Objective Recovery Characteristics

#### Cooling Effects on Blood Plasma Markers

**Fig 5:**
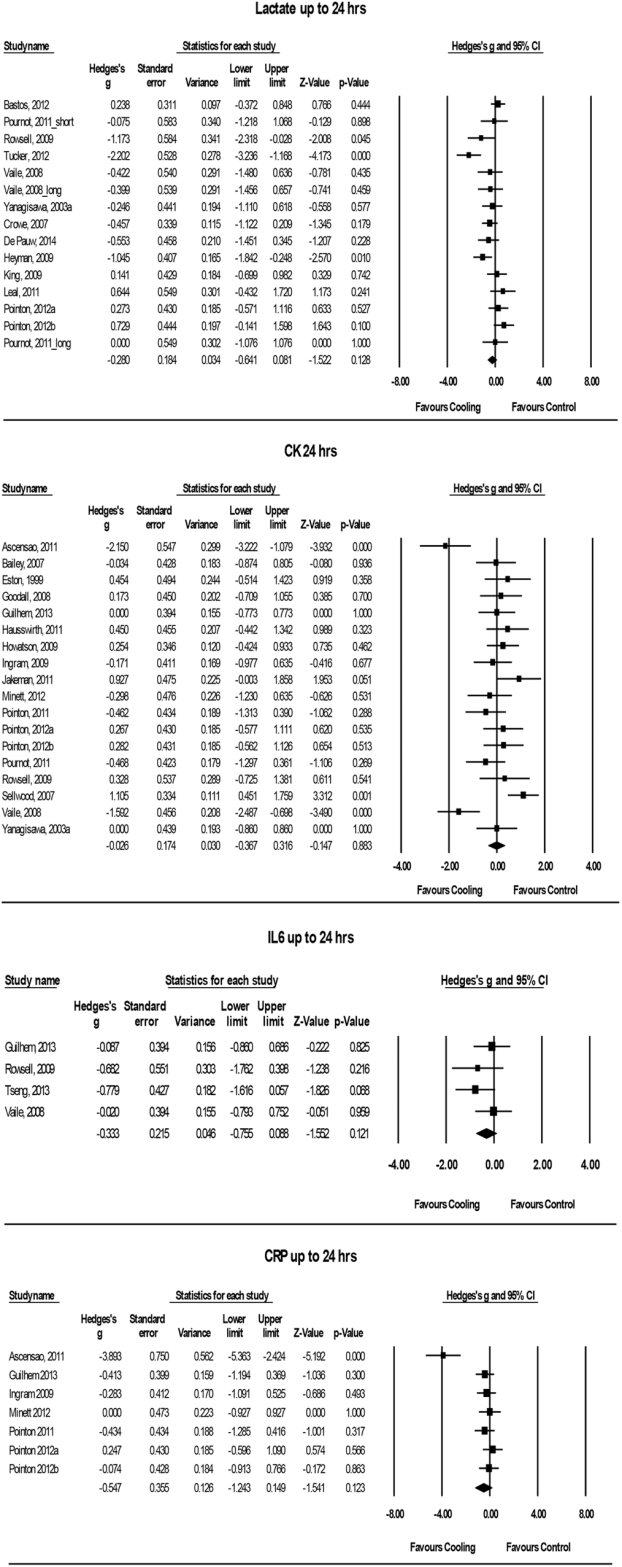
Forest plot of the meta-analysis illustrating the comparison of cooling versus control for measurement of lactate, CK, IL-6 and CRP. CK = creatine-kinase, IL-6 = interleukine-6, CRP = C-reactive protein.

The effect of post-exercise cooling on various blood plasma markers was analysed in 26 studies [2, 4–12, 14, 18–22, 24, 27–29, 31, 33, 35–37, 50]. From those, 13 studies measured lactate-levels [5, 7, 9, 12, 22, 28, 29, 33, 35–37, 50] and 19 measured CK-levels [2, 4, 6–8, 10, 11, 14, 18–21, 24, 27–29, 31, 33, 35]. Seven studies measured both lactate- and CK-levels [7, 9, 27–29, 33, 35]. Detailed information about the results of the meta-analysis of the objective recovery variables can be observed in Fig 5.

#### Cooling Effects on Lactate-Levels

Eight studies analysed blood lactate-levels [5, 9, 22, 27, 28, 36, 37, 50], four studies LDH-levels [7, 29, 33, 35] and one study muscle lactate-levels [12]. Data, measured at follow-up times up to 24 hrs and exactly 24 hrs after the exhaustive exercise, was pooled. Within the first 24 hrs of recovery, the volunteers in the cryotherapy group did not show significant differences compared to the control group. (Hedges’ g: -0.28, 95% CI: -0.64 to 0.08; Cochran’s Q: 33.6, df (Q): 14, p = 0.002; I^2^: 58.3%). The classic fail and safe model analysis suggested, that 6 studies would be needed to bring alpha above the 5% level, indicating a potential risk of publication bias. Subgroup analysis according to time (< 24 hrs vs. 24 hrs) and sex could not explain the heterogeneity. However, the cooling temperature and also the diagnostic procedure to obtain blood lactate or muscle lactate results could significantly explain parts of the heterogeneity (p < 0.001, p = 0.003). Tucker et al. (2012) were the only authors who analysed the lactate-levels by measuring local muscle lactate [12]. After performing the subgroup analyses, one could observe, that only this study showed significant effects, favouring cooling compared to blood lactate and LDH measurements (Hedges’ g: -2.20, 95% CI: -3.24 to -1.17; Cochran’s Q: 0, df (Q): 0.0, p = 1.0; I^2^: 0%). A meta-regression according to cooling temperature showed no significant differences between cooling and alleviated lactate-levels during the 24 hrs recovery time (Slope = 0.04, 95% CI: -0.00 to 0.08, p = 0.079).

#### Cooling Effects on CK-Levels

The possible effects of post-exercise cooling on the CK-levels was investigated in 19 studies up to 72 hrs [2, 4, 6–8, 10, 11, 14, 18–21, 24, 27–29, 31, 33, 35]. The pooled data showed no significant results, favouring cooling compared to the control condition during the 72 hrs recovery time (24 hrs: Hedges’ g: -0.002, 95% CI: -0.33 to 0.32; Cochran’s Q: 49.7, df (Q): 18, p < 0.001; I^2^: 63.8%; 48 hrs: Hedges’ g: -0.19, 95% CI: -0.46 to 0.08; Cochran’s Q: 18.4, df (Q): 13, p = 0.143; I^2^: 29.4%; 72 hrs: Hedges’ g: -0.10, 95% CI: -0.46 to 0.27; Cochran’s Q: 10.3, df (Q): 7, p = 0.175; I^2^: 31.7%). The subgroup analyses could not significantly explain the high heterogeneity, which was only found after 24 hrs of recovery. A meta-regression according to cooling temperature was performed at 24 hrs. The results showed no significant slope between cooling temperature and Hedges’ g (Slope = -0.003, 95% CI: -0.01 to 0.00, p = 0.388). After excluding the extreme low temperatures, presented in the study from Hausswirth et al. (2011), the results of the meta-regression still did not change significantly (Slope < -0.000, 95% CI: -0.01 to 0.01, p = 0.992) [8]. No publication bias could be observed during the visual analysis of the funnel plot.

#### Cooling Effects on Blood Plasma Cytokines

Ten studies used plasma cytokines (IL-6 and CRP)–amongst other–as a recovery variable [2, 4, 7, 10, 11, 21, 24, 27, 28, 33]. Four studies measured the IL-6 levels up to 72 hrs [7, 10, 11, 33] while seven studies measured the CRP levels up to 48 hrs [2, 4, 10, 21, 24, 27, 28].

There were no significant main effects, favouring cooling compared to the control conditions during the 72 hrs recovery period, according to the IL-6 levels (24 hrs: Hedges’ g: -0.33, 95% CI: -0.76 to 0.09; Cochran’s Q: 2.5, df (Q): 3, p = 0.473; I^2^: 0%; 48 hrs: Hedges’ g: -0.35, 95% CI: -0.85 to 0.15; Cochran’s Q: 0.9, df (Q): 2, p = 0.646; I^2^: 0%; 72 hrs: Hedges’ g: -0.43, 95% CI: -0.93 to 0.07; Cochran’s Q: 0.54, df (Q): 2, p = 0.764; I^2^: 0%). Similar results could be observed for the CRP values. No significant results favouring cooling compared to the control conditions could be observed during the 24 hrs recovery period (24 hrs: Hedges’ g: -0.55, 95% CI: -1.24 to 0.15; Cochran’s Q: 25.3, df (Q): 6, p < 0.001; I^2^: 76.3%). The meta-regression for cooling temperature showed no significant slope after 24 hrs of recovery (Slope < 0.000, 95% CI: -0.70 to -0.03, p = 0.032). However, there were significant differences after 48 hrs of recovery, favouring cooling compared to the control condition (48 hrs: Hedges’ g: -0.73, 95% CI: -1.38 to -0.08; Cochran’s Q: 6.9, df (Q): 3, p = 0.075; I^2^: 56.5%). A subgroup analysis according to cooling modality or cooling temperature was not possible because a meaningful grouping was not applicable.

## Discussion

### Quality of Evidence and Limitations

The majority of the studies had a high or unclear risk of bias, which made the interpretation of the results uncertain. The main bias was produced by means of the blinding procedure. The possibilities for blinding cooling applications stay limited. Another problem was the description of the random sequence generation and the procedure of allocation concealment. The power of the selected articles was mainly small according to the individual study population. Bleakly et al. (2012) examined the effects of CWI for preventing and treating muscle soreness after exercise and reported similar limitations according to the individual study population [3]. Another limitation of this composed article is variation in exercise protocols: exercise induced muscle damage protocols varied over sprint- and endurance protocols to various sports protocols. A subgroup analysis according to the various exercise protocols was considered, but not possible because of the wide range of performed exercise protocols. The processed studies used only female, only male or both (female and male) participants. The sex specific factor might have influenced the results in the selected studies. In this context it can be advised to take the amount of adipose tissue over the affected cooling area into account, because it can significantly affect the rate of intramuscular cooling [51]. These findings are important for further studies because the effect of cooling could be highly influenced by the amount of adipose tissue. One needs to evaluate if there is a possibility to create an algorithm for calculating the ideal cooling temperature, respectively cooling duration for specific amounts of adipose tissue. Further studies, which evaluate the effect of cooling, should at least consider the body compositions of the selected study participants. However, when performing the sex specific subgroup analyses across this study, significant differences could only be observed for DOMS up to 48 hrs, favouring men compared to women for cooling applications. The authors of this study are aware of the observed heterogeneity between the studies. The heterogeneity during the meta-analyses was analysed by performing subgroup analyses, sensitivity analyses and meta-regression analyses. However, these analyses according to cooling modality, sex and cooling temperature could not, or only partly explain the high heterogeneity. The various exercise protocols and also the different passive non-cooling recovery strategies, used in the included studies, may be additional factors that lead to the heterogeneity. Subgroup analyses were considered, but a meaningfully grouping according to these factors was not possible. The authors of this composed article are aware that publication bias might occur, due to the fact, that no gray literature was analysed.

### Subjective Recovery Characteristics

One main finding of this study was, that cold therapy (CWI) significantly alleviated the symptoms of DOMS 24 hrs, 48 hrs and 96 hrs after the cooling application. This significant effect was not present at 72 hrs after the cooling application. By performing subgroup analysis, sensitivity analyses and meta-regressions, we could not detect the reason for this non-significant result. However, Tseng et al. (2013) showed a trend, favouring the sham application compared to cooling application [11]. These authors did not mention the cooling temperature of the applied cold pack or the size of the affected skin area. CWI was the most used cooling application across the studies and showed the best effect between cold air, cold pack and WBC. It should be considered, that the treated surface of the body might influence the cooling results. Grahn et al. (2009) showed that the effect of treating multiple body surfaces was additive [52]. Montgomery et al. (2008) included stretching protocols in their control conditions. This active therapy form could have influenced the results [25]. The results of the studies from Tseng et al. (2013) and Montgomery et al. (2008) might be the reasons for the non-significant effects of cooling compared to the control condition in this meta-analysis for DOMS 72 hrs. Leeder et al. (2012) and Bleakley et al. (2012) conducted a meta-analysis for CWI on DOMS. [3, 40]. The results of these authors are in line with the findings of this composed article and confirm the conclusion from Grahn et al. (2009). Furthermore, in this composed article, a step forward was made according to the question from earlier researchers. These researchers concluded, that further studies are needed to support the hypothesis that cryotherapy for well-trained and high-trained individuals is effective to alleviate the symptoms of DOMS during the first 48 hrs of recovery [44]. Another finding was, that cooling significantly reduced the RPE after 24 hrs of recovery. It seems that cooling positively affects the local and overall subjective ratings. In the results of the study and population characteristics could be observed, that n = 8 articles were included with well-trained/elite athletes. The results of these athletes were analysed together with the results of the healthy / trained volunteers. All well-trained volunteers showed results that favoured cooling compared to the control conditions. This was true for DOMS 24 hrs and 48 hrs and RPE 24 hrs and 48 hrs, where these volunteers were present. However, it has to be mentioned, that the results favouring cooling compared to the control condition for RPE 48 hrs were only significant when using the fixed model analysis (Hedges’ g: -0.75, 95% CI: -1.27 to -0.23; Cochran’s Q: 6.23, df (Q): 2, p = 0.044; I^2^: 67.88%). A possible explanation could be that the well-trained volunteers can provide more power and more DOMS and RPE than the normal trained volunteers. These findings can be useful for physiotherapists, coaches and physicians who work with athletes. Especially athletes, who perform exhaustive exercises or sports and possess only short recovery periods between training sessions or competitions, could benefit from these findings.

### Objective Recovery Characteristics

Cooling did not affect objective recovery variables such as lactate-levels, CK-levels or IL6-levels. However, significant differences could be observed favouring cooling at CRP 48 hrs. Because of the high risk of bias according to the blinding procedures and the unclear selection bias, it is not certain that these results represent the real effects of cooling at this point. Furthermore, only four studies with a total of n = 65 volunteers represent this significant outcome. No inflammatory item was significantly lower at any time in this meta-analysis except the CRP value measured at 48 hrs, which made this result questionable. Although the effect size was moderate (Hedges’ g: -0.72), a high heterogeneity could be observed (I^2^: 56.52%) according to Cohen et al. (1992) and Higgins et al. (2002), which could not be explained by meta-regressions [47, 48]. An interesting result could be observed in the subgroup analysis according to the lactate measurement procedure. Muscle-lactate measurement showed significantly lower lactate-levels compared to LDH- and blood lactate measurements. These findings might indicate that muscle- and blood lactate measurements differ from each other. Another possible explanation for the different results can be, that the muscle lactate measurement represents a local effect, while the blood lactate measurement represents a dilution effect of the non-involved muscles. Furthermore, the authors of this composed article are aware that LDH is also used in the literature as an indicator for muscle damage, together with the determination of CK-levels [53, 54]. The “American Association for Clinical Chemistry” shows, that measuring LDH-level is a non-specific test that may be used in the evaluation of different diseases, and that temporary elevations after strenuous exercises are possible [55]. The clinical relevance of lactate-levels as indicators of physical recovery is questionable. However, the overall effect of cooling compared to the control condition for reducing lactate-levels was only significantly different at 24 hrs post-exercise, when using the fixed model analysis (Hedges’ g: -0.244, 95% CI: -0.472 to -0.016; Cochran’s Q: 33.57, df (Q): 14, p = 0.002; I^2^: 58.30%). All meta-regressions according to cooling temperature indicated, that the cooling temperature did not correlate to Hedges’ g. Especially very low temperatures (-110°) which were used in cold chambers, seem to negatively influence the effect of cooling. One needs to consider that very low cooling temperatures were only used for a short period or without direct skin contact. Gregson et al. (2011) recently showed that CWI induced a reduction of femoral artery blood flow and muscle temperature after a 10 min (8°C to 22°C) immersion [56]. From these results one could conclude, that the blood plasma markers and the blood plasma cytokines would change due to blood flow and temperature differences. In this composed articles, neither the blood plasma markers nor the blood plasma cytokines significantly changed between cooling and the control conditions. More studies with a minimum risk of bias are needed to be able to explain the real physiological mechanisms of cold therapy. Moreover, further studies should clarify the real physiological effect, or if the effect of cooling simply reflects the placebo effect. The phenomenon of the placebo effect can easily occur due to the fact that cooling applications are used from many famous athletes during public events. Broatch et al. (2014) recently published results which indicate that an administered placebo immersion is superior in the recovery of muscle strength over 48 hrs compared to TNI and is as effective as CWI. The authors concluded that this result can be attributed to improved subjective ratings, suggesting that the commonly hypothesized physiological benefits surrounding CWI are at least partly placebo related [57]. WBC and cold air applications probably do not cool as deep as cold applications with direct contact to the skin, but only affect skin temperature. Bleakley et al. (2012) recently indicated the possibility, that cryotherapy techniques used in humans do not sufficiently cool muscle tissue to produce any physiological effect [58]. However, the effect of cooling has not been clearly established despite the large volume of research in this area. Another proposed mechanism of cold therapy is a cold-induced nerve-conduction adaption. Herrera et al. (2010) recently showed, that CWI changed the sensory conduction at a physiological level that is sufficient to induce a hypoalgesic effect. However, negative effects of cooling, like inhibition of healing due to inhibition of inflammatory responses and healing related signalling are reported. [41, 43, 59]. Tiidus et al. (2015) recently concluded that cryotherapy or icing, as currently practiced, will not likely be successful in cooling human muscle sufficiently to have any significant influence on muscle repair regardless of the degree of injury. Based on studies in animal models, it may be that if sufficient muscle cooling could be achieved in humans, it could actually delay repair and increase muscle scarring following recovery from significant muscle damage [60].

### Future Study

Future studies should consider a well described random sequence generation and allocation concealment. Although a blinding in cooling studies is difficult, we would advise to blind at least the assessor or even the volunteer by using thermo-neutral applications and not only passive recovery modalities. For the volunteer, the aim of the study is easy to foresee when he/she receives one cooling application and/or one passive recovery strategy. Furthermore, the individual study population was low. Future researchers should include a larger study population with respect to the sex. The selected articles mainly used male participants. More studies with female participants are needed to evaluate, if there is a sex specific difference for cooling effects and how the amount of adipose tissue influence the effects of cooling. We had to spend much time for extracting data out of graphs or to contact researchers to obtain their data. Future studies should present their data for example as mean ± SD and not only present graphs without detailed description. Furthermore, the scaling system for rating DOMS and RPE showed a wide range. Future studies should consider their rating scales and use sensitive and common scaling systems. The extraction of data from graphs implies a potential risk of under- or overestimation of the individual treatment effect. Further studies should consider the use of cheap and mobile cooling equipment, because it might be clinically more relevant for an advisor to use such devices compared to fixed and/or expensive cooling devices.

## Conclusion

From the current results, one can conclude, that cooling is superior compared to passive recovery strategies after various exhaustive or muscle damaging exercise protocols. These results relate to the subjective effects of different cooling applications. Cooling showed significant effects in reducing the symptoms of DOMS (up to 96 hrs) and RPE (up to 24 hrs) compared to passive control interventions. CWI achieved the best effect with respect to the other cooling applications. To sum up the results of the individual studies: the mean temperature of the studies, showing a significant result favouring cooling compared to the passive recovery intervention, was 10°C (range: 5°C to 13°C). The reported and suggested cooling time for alleviating the subjective symptoms is 13 min (range: 10 min to 24 min). Cooling did not significantly affect the objective recovery outcomes compared to passive control interventions. Despite the detailed analysis of the individual study results, it must be viewed with caution, because the risk of bias was high according to the blinding procedures and remained unclear for the selection bias. The between-studies heterogeneity could be partly explained. Other factors (various pre-cooling exercises and various passive non-cooling recovery strategies) might influence the different recovery characteristics.

## Supporting Information

S1 PRISMA Checklist (PDF)
